# Association of Emergency Clinicians' Assessment of Mortality Risk With Actual 1-Month Mortality Among Older Adults Admitted to the Hospital

**DOI:** 10.1001/jamanetworkopen.2019.11139

**Published:** 2019-09-13

**Authors:** Kei Ouchi, Tania Strout, Samir Haydar, Olesya Baker, Wei Wang, Rachelle Bernacki, Rebecca Sudore, Jeremiah D. Schuur, Mara A. Schonberg, Susan D. Block, James A. Tulsky

**Affiliations:** 1 Department of Emergency Medicine, Brigham and Women’s Hospital, Boston, Massachusetts; 2 Department of Emergency Medicine, Harvard Medical School, Boston, Massachusetts; 3 Serious Illness Care Program, Ariadne Labs, Boston, Massachusetts; 4 Department of Emergency Medicine, Maine Medical Center, Portland, Maine; 5 Division of Sleep Medicine, Department of Medicine, Brigham and Women’s Hospital, Boston, Massachusetts; 6 Department of Psychosocial Oncology and Palliative Care, Dana-Farber Cancer Institute, Boston, Massachusetts; 7 Division of Palliative Medicine, Department of Medicine, Brigham and Women’s Hospital, Boston, Massachusetts; 8 Department of Medicine, University of California, San Francisco; 9 Department of Emergency Medicine, Alpert Medical School of Brown University, Providence, Rhode Island; 10 Department of Medicine, Beth Israel Deaconess Medical Center, Boston, Massachusetts; 11 Department of Psychiatry, Brigham and Women’s Hospital, Boston, Massachusetts

## Abstract

**Importance:**

The accuracy of mortality assessment by emergency clinicians is unknown and may affect subsequent medical decision-making.

**Objective:**

To determine the association of the question, “Would you be surprised if your patient died in the next one month?” (known as the *surprise question*) asked of emergency clinicians with actual 1-month mortality among undifferentiated older adults who visited the emergency department (ED).

**Design, Setting, and Participants:**

This prospective cohort study at a single academic medical center in Portland, Maine, included consecutive patients 65 years or older who received care in the ED and were subsequently admitted to the hospital from January 1, 2014, to December 31, 2015. Data analyses were conducted from January 2018 to March 2019.

**Exposures:**

Treating emergency clinicians were required to answer the surprise question, “Would you be surprised if your patient died in the next one month?” in the electronic medical record when placing a bed request for all patients who were being admitted to the hospital.

**Main Outcomes and Measures:**

The primary outcome was mortality at 1 month, assessed from the National Death Index. The secondary outcomes included accuracies of responses by both emergency clinicians and admitting internal medicine clinicians to the surprise question in identifying older patients with high 6-month and 12-month mortality.

**Results:**

The full cohort included 10 737 older adults (mean [SD] age, 75.9 [8.8] years; 5532 [52%] women; 10 157 [94.6%] white) in 16 223 visits treated in the ED and admitted to the hospital. There were 5132 patients (31.6%) with a Charlson Comorbidity Index score of 2 or more. Mortality rates were 8.3% at 1 month, 17.2% at 6 months, and 22.5% at 12 months. Emergency clinicians stated that they would not be surprised if the patient died in the next month for 2104 patients (19.6%). In multivariable analysis controlling for age, sex, race, admission diagnosis, and comorbid conditions, the odds of death at 1 month were higher in patients for whom clinicians answered that they would not be surprised if the patient died in the next 1 month compared with patients for whom clinicians answered that they would be surprised if the patient died in the next 1 month (odds ratio, 2.4 [95% CI, 2.2-2.7]; *P* < .001). However, the diagnostic test characteristics of the surprise question were poor (sensitivity, 20%; specificity, 93%; positive predictive value, 43%; negative predictive value, 82%; accuracy, 78%; area under the receiver operating curve of the multivariable model, 0.73 [95% CI, 0.72-0.74; *P* < .001]).

**Conclusions and Relevance:**

This study found that asking the surprise question of emergency clinicians may be a valuable tool to identify older patients in the ED with a high risk of 1-month mortality. The effect of implementing the surprise question to improve population-level health care for older adults in the ED who are seriously ill remains to be seen.

## Key Points

**Question:**

What is the association of emergency clinicians’ assessment of mortality risk with the actual 1-month mortality among older adults who are admitted to the hospital from the emergency department?

**Findings:**

In this prospective cohort study including 10 737 older adults who visited the emergency department, emergency clinicians’ response of no to the question, “Would you be surprised if your patient died in the next one month?” was associated with 1-month mortality after controlling for confounders. However, the diagnostic test characteristics of the surprise question were poor.

**Meaning:**

Asking emergency clinicians the surprise question may be a valuable tool to identify older patients in the ED with high risk of 1-month mortality.

## Teaser

This cohort study examines the accuracy of clinicians’ assessments of mortality risk among older adults admitted to the hospital from the emergency department compared with the actual 1-month mortality of these patients.

## Introduction

Approximately 75% of older adults with serious, life-limiting illnesses visit the emergency department (ED) during the final 6 months of life.^1^ Emergency department visits often mark an inflection point in a patient’s illness trajectory, signaling a more rapid rate of decline.^2,3^ Many patients have not formulated their goals for care in the context of their serious illness,^4^ and approximately 56% to 99% of patients do not have documentation of such goals available at the time of ED presentation.^5^ Most patients who are seriously ill have priorities other than simply living as long as possible,^6^ yet they are at risk of receiving care that does not align with their goals.^7^

Conversations about serious illness care goals (ie, serious illness conversation) are associated with lower rates of in-hospital death, less aggressive medical care at the end of life, earlier hospice referrals, increased peacefulness, and a 56% greater likelihood of having end-of-life wishes known and followed.^8,9,10,11,12,13,14,15,16,17^ Furthermore, patients with documented serious illness conversations experience a 36% reduction in the cost of end-of-life care, with an mean cost savings of $1041 per patient in the final week of life.^18^ A study by Smith et al^19^ reported that earlier serious illness conversations are 1 of 5 key changes that can reduce the costs for health care. Yet only approximately 37% of older adults who are seriously ill have these conversations with their physicians,^9^ and it is often late in the disease course (33 days before death^20^).

Emergency physicians recognize that an ED visit provides a time and a location for older adults who are seriously ill to discuss serious illness care goals.^21^ For patients who are seriously ill but clinically stable and who are likely to experience a decline in illness trajectory, engaging them in such a discussion in the ED may be an ideal moment to facilitate serious illness conversations.^22^ Introduction or reinitiation of serious illness conversations before these patients become clinically unstable may be acceptable and feasible in the ED.^23^ The current clinical practice is constrained by the lack of feasible and reliable approaches to identify patients with limited life expectancy who are most likely to benefit from serious illness conversations after an ED visit. A practical method is needed to help emergency clinicians identify patients who are seriously ill and at the highest risk of mortality and to ensure that such patients receive serious illness conversations after being admitted to the hospital. The so-called surprise question, worded as “Would you be surprised if this patient died in the next 12 months?” is a method to obtain the clinician’s overall clinical impression associated with the actual 12-month mortality among patients with a life-limiting illness. The surprise question has demonstrated sensitivity of 21% to 84% and specificity of 51% to 94% in prior studies^24,25,26,27,28,29,30,31^ among outpatient patients with advanced cancer or chronic kidney disease undergoing hemodialysis and may be uniquely valuable in the time-pressured ED setting to identify older patients with high risk of mortality.^32^ The surprise question may help emergency clinicians to identify older adults who are seriously ill and who most urgently require serious illness conversation on admission so that no such patients continue the hospitalization without formulating serious illness care goals. However, to our knowledge, previous studies of the surprise question are limited by their small sample size, variable magnitudes of association, and focus on specific disease populations, limiting the generalizability of this method.

In this large cohort study, we aimed to prospectively test the association of the surprise question with the actual 1-month mortality among a diverse population of undifferentiated older patients in the ED. We propose that emergency clinicians’ ability to estimate prognosis is the most accurate in the short term (ie, 1-month vs the traditional 12-month mortality) by the nature of their clinical practice. To most effectively devote limited resources in a time-pressured environment, the short-term prognosis for patients presenting to the ED is important to identify those who most urgently require serious illness conversations.

## Methods

### Study Design

We conducted a prospective cohort study to examine the association of physicians’ response to the surprise question with the actual 1-month mortality of patients who visited the ED at an academic, urban hospital with an annual volume of 70 000 visits, including 24% by patients aged 65 years and older and a hospital admission rate of 47% among these older patients. The study protocol was approved by the Partners Healthcare institutional review board as human subject research. Informed consent was waived because there was no interaction with patients and their risk was strictly the potential breach of confidentiality, which was deemed a minimal risk. This study is reported following the Strengthening the Reporting of Observational Studies in Epidemiology (STROBE) reporting guideline. Data analyses were conducted from January 2018 to March 2019.

### Participants and Procedures

We included all patients 65 years and older who received care in the ED and were admitted to the hospital from January 1, 2014, to December 31, 2015. We chose to include older adults who were admitted to the hospital because they would be more likely to have higher mortality compared with those who were discharged and therefore were the most appropriate patient population to study to identify older adults with the highest 1-month mortality in the ED. The patients who were discharged were more likely to live beyond 1 month, and the treating emergency clinicians were more likely to report that they would be surprised if the patient died in the next 1 month. Therefore, including the discharged patients may have risked overinflating the overall accuracy of the clinical impression. We excluded patients who were admitted to the hospital without going through the ED and those who were transferred from the ED to the cardiac catheterization laboratory or operating room after the ED (ie, expected to have high mortality).

When placing a bed request in the electronic medical record (EMR) for any patient being admitted to the hospital, emergency clinicians were required to answer a mandatory question, “Would you be surprised if your patient died in the next one month?” Placing a bed request in the EMR is a requirement to admit a patient into the hospital from the ED. Enrollment took place consecutively 7 days per week, 24 hours per day. We obtained the death records from the National Death Index (NDI), the central computerized database containing all certified deaths in the United States, on January 1, 2018. We used the complete NDI data from 2014 to 2015 and early release data from January to December 2016, which contained more than 90% of the 2016 data. Mortality information was ascertained by matching the cohort data set from the EMR to NDI death certificate records. We used the Social Security number, first and last names, and date of birth to match the records. Participants were considered a match when all 3 variables were completely matched. Participants were assigned a vital status code (0 = assumed alive; 1 = assumed deceased) based on their status as of 1 month from the time they visited the ED. Participants with incomplete matches or multiple NDI-matched death records were excluded from the study.

### Variables

Our primary outcome was the accuracy of clinicians’ response to the surprise question in identifying older patients in the ED with 1-month mortality. Our secondary outcomes included the accuracies of responses by both emergency clinicians and admitting internal medicine clinicians to the surprise question in identifying older patients with high 6- or 12-month mortality. We controlled for the following potential confounders in our analysis: patient demographic information (ie, age, sex, and self-reported race), Charlson Comorbidity Index score,^33^ source of ED arrival (eg, home, another hospital, nursing facility), and principal diagnosis using Clinical Classifications Software.^34^ These variables were directly extracted from the EMR as structured data variables. No manual EMR abstraction was conducted. Emergency clinicians commonly consider many clinical variables (eg, vital signs) to derive their overall clinical impressions (ie, the response to the surprise question). Some potentially confounding information may not have been considered immediately (eg, comorbid conditions); thus, we decided to include such additional variables in the multivariable model. We did not include other potential confounders easily accessible to the clinician when determining the overall clinical impression, such as vital signs, laboratory values, and other clinical scoring systems (eg, Acute Physiologic Assessment and Chronic Health Evaluation). Many, if not all, of these variables should be incorporated into clinicians’ overall clinical impression.

### Statistical Analysis

Patients were classified into 2 subgroups based on how clinicians answered the surprise question: (1) “No, I would not be surprised if this patient died in the next one month,” and (2) “Yes, I would be surprised if this patient died in the next one month.” A generalized estimating equation model with binary outcome was used to estimate the association of death and the response to the surprise question along with other potential covariates to take into account the possible correlation between multiple visits from the same patient. We started fitting a bivariate generalized estimating equation model with a pool of potential covariates that may be clinically relevant to the outcome 1 at a time. Covariates with significant associations were included in the final multivariable model. We determined the sensitivity, specificity, positive and negative predictive values, accuracy, odds ratio (OR), and area under the receiver operating curve at 1 month using SAS statistical software version 9.4 (SAS Institute). *P* values were 2-tailed, and statistical significance was set at *P* less than .05. We presented the overall difference in 1-month survival between the 2 subgroups using Kaplan-Maier curves. Based on the study by Lilley et al,^27^ we expected the accuracy to be at least 0.60. To achieve the precision of at least 0.02, we hypothesized that we would require at least 10 000 patients for the sample size. We also performed a sensitivity analysis to account for intensive care unit admission status.

## Results

**Figure 1.**
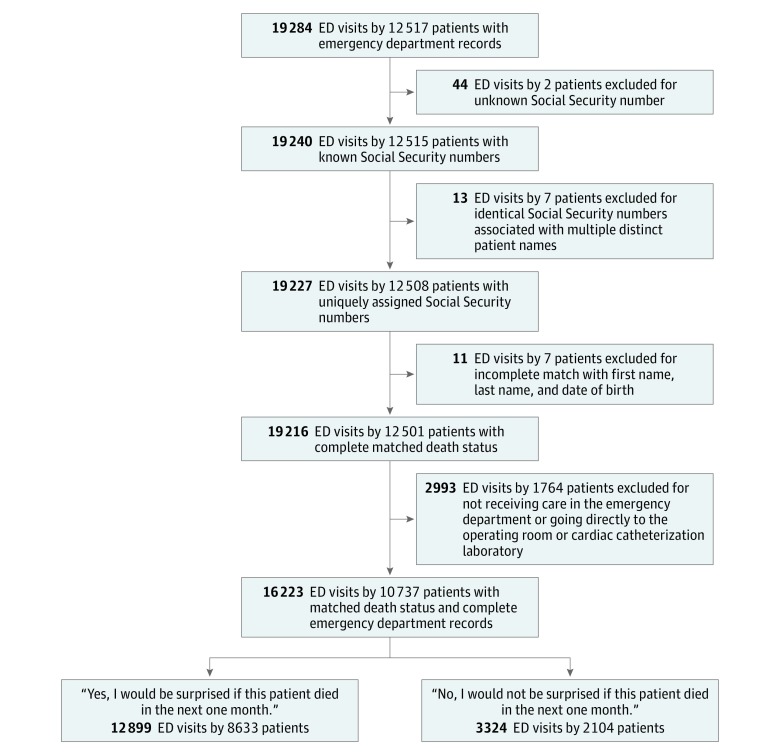
Cohort Selection Flowchart ED indicates emergency department.

We identified 19 284 ED visits by 12 517 patients from January 1, 2014, to December 31, 2015. Sixteen patients were excluded owing to unknown Social Security numbers, inconsistent Social Security number matches to the NDI records, or incomplete matches of the name, date of birth, or Social Security number. We also excluded 1764 patients because they were transferred directly to the cardiac catheterization laboratory or operating room. We identified 16 223 ED visits by 10 737 patients in our final cohort (Figure 1). The patients had a mean (SD) age of 75.9 (8.8) years and included 5532 women (51.5%), 10 157 patients (94.6%) who were white, and 5132 patients (31.6%) with a Charlson Comorbidity Index score of 2 or more (Table 1). The mortality rates were 8.3% at 1 month, 17.2% at 6 months, and 28.5% at 12 months. Eighty-five ED clinicians answered the surprise question, including 33 attending-level emergency physicians (mean time in practice, 9.2 years; 67% men), 40 resident-level emergency physicians (mean time in practice, 2.1 years; 74% men), and 12 physician assistants (mean time in practice, 9.2 years; 58% women). Within this group, the mean admission rate was 47% (range, 44%-50%) for their patients 65 years and older.

**Table 1.** Cohort Demographic and Admission Characteristics

Characteristic	No. (%)
**Patient Characteristics**	
Individual patients, No.	10 737
Age, mean (SD), y	75.9 (8.7)
Sex	
Men	5205 (48.5)
Women	5532 (51.5)
Race	
White	10 157 (94.6)
Black	62 (0.6)
Asian	78 (0.7)
Not reported	354 (3.3)
Mortality, mo	
1	892 (8.3)
6	1846 (17.2)
12	2412 (22.5)
Repeated ED visits	3059 (28.5)
**Admission Characteristics**	
Individual visits, No.	16 223
Most common admission diagnoses	
Shortness of breath	1190 (7.3)
Chest pain	512 (3.2)
Fever	446 (2.8)
Altered mental status	347 (2.1)
Syncope or collapse	302 (1.9)
Congestive heart failure	266 (1.6)
Pneumonia	242 (1.5)
Stroke	222 (1.4)
Atrial fibrillation	189 (1.2)
Malaise or fatigue	180 (1.1)
Source of ED arrival	
Home	11 009 (67.9)
Physician’s office	1535 (9.5)
Transfer from a nursing home	1037 (6.4)
Transfer from another hospital	1192 (7.4)
Other	1450 (8.9)
Charlson Comorbidity Index score	
0	8958 (55.2)
1	2133 (13.2)
≥2	5132 (31.6)

Abbreviation: ED, emergency department.

**Figure 2.**
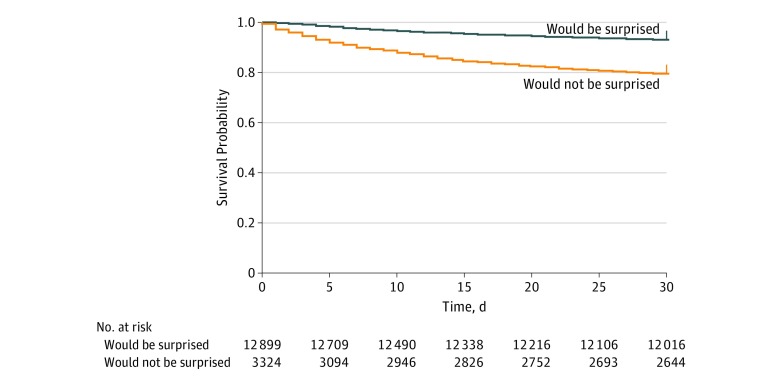
One-Month Survival Curves Orange line indicates patients for whom clinicians responded that they would not be surprised if the patient died in 1 month; blue line, patients for whom clinicians responded that they would be surprised if the patient died in 1 month; crosses, censored data.

Of 10 737 patients, the clinicians stated that they would not be surprised if the patient died in the next 1 month for 2104 patients (19.6%), and 893 of 10 737 patients (8.3%) died within 1 month. In bivariate analysis (Table 2), the odds of death at 1 month in patients for whom clinicians answered that they would not be surprised if the patient died in 1 month was 3.3-fold that of patients for whom clinicians answered that they would be surprised if the patient died in 1 month (OR, 3.3 [95% CI, 3.0-3.7]; *P* < .001). In multivariable analysis controlling for age, sex, race, admission diagnosis, and comorbid conditions (Table 2), the odds of death at 1 month in patients for whom clinicians answered that they would not be surprised if the patient died in 1 month was 2.4-fold that of patients for whom clinicians answered that they would be surprised if the patient died in 1 month (OR, 2.4 [95% CI, 2.2-2.7]; *P* < .001). The surprise question answered by clinicians demonstrated sensitivity of 20% and specificity of 93%. Given the 1-month mortality rate of 8.3% in our cohort, the positive and negative predictive values were 43% and 82%, respectively. The accuracy was 78% (Table 3). The area under the receiver operating curve of the bivariate model was 0.63 (95% CI, 0.61-0.64; *P* < .001), and the area under the receiver operating curve of the multivariable model was 0.73 (95% CI, 0.72-0.74; *P* < .001). The model performance improved from 0.70 to 0.73 by adding the surprise question as a covariate. Among 2104 patients for whom clinicians answered that they would not be surprised the patient died in 1 month, only 533 patients actually died within 1 month (Figure 2). Similar test characteristics were demonstrated by the admitting hospital clinicians. Emergency clinicians’ ability to accurately identify patients who would die vs patients who would survive was consistent at 1, 6, and 12 months after the ED visit (eTable 1 and eTable 2 in the Supplement). There were no statistically significant differences in our sensitivity analysis to account for intensive care unit admission status (eTable 3 in the Supplement). We did not have any missing data for responses to the surprise question or for 1-month mortality.

**Table 2.** Bivariate and Multivariable Analysis for 1-Month Mortality

Variable	Bivariate Analysis		Multivariable Analysis	
	OR (95% CI)	*P* Value	OR (95% CI)	*P* Value
Treating clinician answered no to the surprise question^a^	3.3 (3.0-3.7)	<.001	2.4 (2.2-2.7)	<.001
Age, per year	1.1 (1.0-1.1)	<.001	1.0 (1.0-1.1)	<.001
Women^b^	1.0 (0.9-1.1)	.87	0.9 (0.8-1.0)	.02
Nonwhite race^c^	3.7 (3.2-4.4)	<.001	3.5 (2.9-4.2)	<.001
Most common admission diagnoses				
Shortness of breath	1.3 (1.1-1.6)	.004	1.1 (0.9-1.4)	.19
Chest pain	0.4 (0.3-0.6)	<.001	0.5 (0.3-0.7)	<.001
Fever	1.6 (1.3-2.1)	<.001	1.1 (0.9-1.5)	.39
Altered mental status	1.6 (1.2-2.2)	.003	1.0 (0.7-1.4)	.90
Syncope or collapse	0.5 (0.3-0.8)	.006	0.4 (0.2-0.8)	.003
Congestive heart failure	1.2 (0.8-1.9)	.40	0.7 (0.5-1.2)	.23
Pneumonia	1.8 (1.2-2.6)	.006	1.0 (0.7-1.5)	.99
Stroke	1.6 (1.1-2.3)	.02	1.2 (0.8-1.7)	.39
Atrial fibrillation	0.8 (0.5-1.3)	.28	0.7 (0.4-1.2)	.15
Malaise or fatigue	1.3 (0.9-1.9)	.18	1.0 (0.7-1.5)	.93
Other	1 [Reference]	NA	1 [Reference]	NA
Source of ED arrival				
Home	1 [Reference]	NA	1 [Reference]	NA
Physician’s office	0.7 (0.6-0.9)	.004	0.8 (0.7-1.0)	.06
Transfer from a nursing home	2.8 (2.4-3.3)	<.001	2.0 (1.6-2.3)	<.001
Transfer from another hospital	0.9 (0.8-1.2)	.64	1.1 (0.9-1.4)	.48
Other	2.3 (2.0-2.7)	<.001	1.8 (1.5-2.1)	<.001
Charlson Comorbidity Index score				
0	1 [Reference]	NA	1 [Reference]	NA
1	0.9 (0.8-1.1)	.50	0.7 (0.6-0.9)	<.001
≥2	1.8 (1.6-2.0)	<.001	1.3 (1.2-1.5)	<.001

Abbreviations: ED, emergency department; NA, not applicable; OR, odds ratio.

^a^ At the time of requesting a bed through the electronic medical record system for the patient to be admitted to the hospital, the treating clinician was required to answer the surprise question, “Would you be surprised if your patient died in the next one month?” Comparison is answering, “No, I would not be surprised,” vs “Yes, I would be surprised.”

^b^ Compared with men.

^c^ Compared with white race.

**Table 3.** Diagnostic Test Characteristics of the Surprise Question Asked of Emergency Clinicians for the Actual 1-Month Mortality^a^

Characteristic	Patient Vital Status at 1 mo From ED Visit			
	Deceased	Alive	Total, No. (%)	Test Characteristic
Clinician response to the surprise question^b^				
No, I would not be surprised	685^c^	2639^d^	3324 (20.5)	Sensitivity: 0.20
Yes, I would be surprised	896^d^	12 003^c^	12 899 (79.5)	Specificity: 0.93
Total, No. (%)	1581 (9.8)	14 642 (90.3)	16 223 (100)	
Predictive values	PPV: 0.43	NPV: 0.82		Accuracy: 0.78

Abbreviations: ED, emergency department; NPV, negative predictive value; PPV, positive predictive.

^a^ Analysis was performed at individual patient visit level with general estimate equation model to account for repeated visits by the same patients.

^b^ At the time of requesting a bed through the electronic medical record system for the patient to be admitted to the hospital, the treating clinician was required to answer the surprise question, “Would you be surprised if your patient died in the next one month?” Clinicians could respond, “No, I would not be surprised,” or “Yes, I would be surprised.”

^c^ Accurate prediction.

^d^ Inaccurate prediction.

## Discussion

Emergency clinicians asserted that they would not be surprised if 1 in 5 of their older patients in the ED died within the next month. Our study confirms that the emergency clinicians’ overall clinical assessment is associated with patients’ actual mortality (OR, 2.4 [95% CI, 2.2-2.7]; *P* < .001) (Table 2). However, the diagnostic test characteristic of the surprise question alone was poor, which makes it a poor screening tool for identifying patients with high risk of 1-month mortality.

Our findings are consistent with prior studies that demonstrated the association of the clinicians’ overall clinical assessment with outcome in other clinical settings.^26,27,29,30,35,36,37^ Furthermore, our study expanded on the available literature that the overall clinical impressions of emergency clinicians who have no prior knowledge or existing relationships with patients are associated with 1-month mortality among older adults whom the clinicians admit to the hospital. Even in time-pressured ED settings, the surprise question has accuracy comparable with that of previously studied clinical settings (eg, outpatient oncology and outpatient nephrology clinics).^27,28,29^ The magnitude of association in our study was much lower (OR, 2.4) compared with prior studies (OR, 3-11).^27,28,29^ This finding may be due to a larger sample size (improving the statistical precision), higher clinical acuity and needs of patients in the ED (ie, emergency clinicians’ perception of acuity may be blunted, resulting in downward bias on the magnitude of association),^38,39^ and lower study mortality (8.3% vs 6% to 45%^24,26,29,30,31^) compared with prior studies in other clinical settings.

Regardless of the diagnostic test characteristics, the surprise question, with its high specificity, remains an important adjunct for identifying patients who may require serious illness conversations. In the ED, where time is of the essence, quick identification that requires less than a few seconds is valuable and feasible, and the use of the surprise question has been shown to be feasible, acceptable, and easy to use in the ED.^40^ Additionally, engaging treating clinicians to assign their clinical judgment to the patients’ mortality may persuade more clinicians to solicit palliative care consultation or serious illness conversations themselves. The overestimation by the surprise question may be appropriate, since two 2019 studies^16,17^ have shown that there is no harm in introducing serious illness conversations earlier in the disease course. The accuracy we demonstrated of the surprise question may be useful for emergency clinicians to act on their clinical intuition to ensure serious illness conversations occur on hospital admission by communicating with the admitting physicians about a patient’s likely prognosis.

### Limitations

Our study has several limitations. The cohort was established in a single, urban, tertiary care, academic medical center with a predominantly white population, all of which may limit the generalizability of the findings to other clinical settings. However, the clinical characteristics of the patients and clinicians were likely comparable to most EDs in the United States. The small proportion of nonwhite patients in this cohort may have produced an unstable point estimate. The clinicians in this study only made a quick judgment of their patients. Sampling bias could have occurred if the surprise question was asked before clinical deterioration of the patients. Although unlikely to bias our final results in significant ways given such a small proportion of patients in this category (7 of 10 737 patients), the potential effect of excluding patients with incomplete NDI matches is unknown. We were unable to control for potential confounding by Emergency Severity Index, chief concern, length of stay, and uncommon admission diagnoses. Some of this information may have been incorporated into clinicians’ overall clinical assessment of their patients using the surprise question. We did not include other clinicians (eg, nurses) to understand how their overall assessment of the patients may be different from that of the emergency physicians and physician assistants, although we know from a 2001 study^41^ that the assessments of other clinicians may be comparable to those of physicians. Further study may be warranted to investigate whether other members of the clinical team in the ED can reliably answer the surprise question to improve the scalability of implementation.

## Conclusions

This study found that asking emergency physicians and physician assistants the surprise question may be a valuable tool to identify older patients in the ED with high risk of 1-month mortality, enhancing access to appropriate serious illness conversations and palliative care services for this population. The potential effect of implementing the surprise question to improve population-level health care for older adults in the ED who are seriously ill remains to be seen.

## Notes

Supplement. **eTable 1.** Diagnostic Test Characteristics of the Surprise Question Asked of Emergency Physicians Compared With Admitting Internal Medicine Physicians **eTable 2.** Diagnostic Test Characteristics of the Surprise Question for Actual 6-Month and 12-Month Mortality **eTable 3.** Sensitivity Analysis of the Surprise Question Test Characteristics After Excluding Patients Admitted to the Intensive Care Unit
