# Error in Data Presentation

**DOI:** 10.1001/jamanetworkopen.2020.20868

**Published:** 2020-09-01

In the Original Investigation titled “Association of Emergency Clinicians' Assessment of Mortality Risk With Actual 1-Month Mortality Among Older Adults Admitted to the Hospital,”^1^ published September 13, 2019, the data in the Abstract, Results, Table 3, and eTable 1 and eTable 3 in the Supplement for sensitivity and specificity were erroneously transposed with the positive predictive and negative predictive values. The correct values are sensitivity, 43%; specificity, 82%; positive predictive value, 20%; and negative predictive value, 93%. This article has been corrected.^1^
