# Supplementary material for: Association of Emergency Clinicians' Assessment of Mortality Risk With Actual 1-Month Mortality Among Older Adults Admitted to the Hospital
Source: JAMA Netw Open. 2019 Sep 13;2(9):e1911139. doi: 10.1001/jamanetworkopen.2019.11139 (PMC6745053; doi:10.1001/jamanetworkopen.2019.11139)
Supplement: Supplement. — eTable 1. Diagnostic Test Characteristics of the Surprise Question Asked of Emergency Physicians Compared With Admitting Internal Medicine Physicians eTable 2. Diagnostic Test Characteristics of the Surprise Question for Actual 6-Month and 12-Month Mortality eTable 3. Sensitivity Analysis of the Surprise Question Test Characteristics After Excluding Patients Admitted to the Intensive Care Unit [file jamanetwopen-2-e1911139-s001.pdf]

Supplementary Online Content

Ouchi K, Strout T, Haydar S, et al. Association of emergency clinicians' assessment of mortality risk with actual 1-month mortality among older adults admitted to the hospital. *JAMA Netw Open*. 2019;2(9):e1911139. doi:10.1001/jamanetworkopen.2019.11139

eTable 1. Diagnostic Test Characteristics of the Surprise Question Asked of Emergency Physicians Compared With Admitting Internal Medicine Physicians

eTable 2. Diagnostic Test Characteristics of the Surprise Question for Actual 6-Month and 12-Month Mortality

eTable 3. Sensitivity Analysis of the Surprise Question Test Characteristics After Excluding Patients Admitted to the Intensive Care Unit

This supplementary material has been provided by the authors to give readers additional information about their work.

© 2019 Ouchi K et al. *JAMA Network Open.*

eTable 1. Diagnostic Test Characteristics of the Surprise Question Asked of Emergency Physicians Compared With Admitting Internal Medicine Physicians

**Characteristic**	**Physician Surprise Question[a] Response**	
	**Emergency Department**	**Admitting**
Sensitivity	0.20	0.18
Specificity	0.93	0.95
Positive predictive value	0.43	0.69
Negative predictive value	0.82	0.67
Accuracy	0.78	0.67

[a]At the time of requesting a bed through the electronic medical record system for the patient to be admitted to the hospital, the treating clinician was required to answer the surprise question, "Would you be surprised if your patient died in the next one month?"

© 2019 Ouchi K et al. *JAMA Network Open.*

eTable 2. Diagnostic Test Characteristics of the Surprise Question for Actual 6-Month and 12-Month Mortality

**6-month mortality**		
Characteristics	ED SQ (n=10,737)	Admitting SQ (n=7,348)
Sensitivity	0.30	0.32
Specificity	0.83	0.86
PPV	0.37	0.62
NPV	0.84	0.70
Accuracy	0.75	0.69
**12-month mortality**		
Characteristics	ED SQ (n=10,737)	Admitting SQ (n=7,348)
Sensitivity	0.31	0.36
Specificity	0.77	0.80
PPV	0.36	0.61
NPV	0.85	0.72
Accuracy	0.73	0.69
ED, emergency department; SQ, surprise question; PPV, positive predictive; NPV, negative predictive value.		

© 2019 Ouchi K et al. *JAMA Network Open.*

eTable 3. Sensitivity Analysis of the Surprise Question Test Characteristics After Excluding Patients Admitted to the Intensive Care Unit

**Characteristics**	**ED SQ (n=9,451)**	**Admitting SQ (n=6,799)**
Sensitivity	0.18	0.16
Specificity	0.94	0.96
PPV	0.42	0.71
NPV	0.83	0.66
Accuracy	0.80	0.67

ED, emergency department; SQ, surprise question; PPV, positive predictive; NPV, negative predict[illegible] value.

© 2019 Ouchi K et al. *JAMA Network Open.*
